# Parkin Regulates the Activity of Pyruvate Kinase M2*

**DOI:** 10.1074/jbc.M115.703066

**Published:** 2016-03-14

**Authors:** Kun Liu, Fanzhou Li, Haichao Han, Yue Chen, Zebin Mao, Jianyuan Luo, Yingming Zhao, Bin Zheng, Wei Gu, Wenhui Zhao

**Affiliations:** From the ‡ Department of Biochemistry and Molecular Biology, Peking University Health Science Center and Beijing Key Laboratory of Protein Posttranslational Modifications and Cell Function, Beijing 100191, China,; § Ben May Department of Cancer Research, The University of Chicago, Chicago, Illinois 60637,; ¶ Department of Genetics, Peking University Health Science Center, Beijing 100191, China,; ‖ Cutaneous Biology Research Center, Massachusetts General Hospital, Harvard Medical School, Charlestown, Massachusetts 02129, and; ** Institute for Cancer Genetics and Department of Pathology and Cell Biology, College of Physicians and Surgeons, Columbia University, New York, New York 10032

**Keywords:** glycolysis, parkin, Parkinson disease, pyruvate kinase, ubiquitylation (ubiquitination), pyruvate kinase M2

## Abstract

Parkin, a ubiquitin E3 ligase, is mutated in most cases of autosomal recessive early onset Parkinson disease. It was discovered that Parkin is also mutated in glioblastoma and other human malignancies and that it inhibits tumor cell growth. Here, we identified pyruvate kinase M2 (PKM2) as a unique substrate for parkin through biochemical purification. We found that parkin interacts with PKM2 both *in vitro* and *in vivo*, and this interaction dramatically increases during glucose starvation. Ubiquitylation of PKM2 by parkin does not affect its stability but decreases its enzymatic activity. Parkin regulates the glycolysis pathway and affects the cell metabolism. Our studies revealed the novel important roles of parkin in tumor cell metabolism and provided new insight for therapy of Parkinson disease.

## Introduction

Parkin, a RING-HECT hybrid E3 ubiquitin ligase (1), is mutated in most cases of autosomal recessive early onset Parkinson disease, which is a neurodegenerative disease associated with loss of dopaminergic neurons in the midbrain (2, 3). Parkin plays a central role in mitochondrial homeostasis and mitophagy by ubiquitylating and tagging depolarized or damaged mitochondria for clearance (4). Parkin translocates to depolarized or impaired mitochondria from the cytosol (5–7) and ubiquitylates a number of proteins within the mitochondrial outer membrane (8, 9).

Increasing evidence has shown that parkin functions as a tumor suppressor. Human parkin gene localizes at chromosome 6q25-q26 region, which is a common fragile site. It has been found that this region is often deleted in ovarian, lung, and breast cancers (10). Parkin knock-out mice had enhanced hepatocyte proliferation and developed macroscopic hepatic tumors with the characteristics of hepatocellular carcinoma (11). Recently, it was discovered that parkin is mutated in glioblastoma and other human malignancies (12). Cancer-specific mutations abrogate the growth-suppressive effects of the parkin protein. Parkin mutations in cancer decrease its E3 ligase activity, compromising its ability to ubiquitylate cyclin E and resulting in mitotic instability (12). Several studies indicate that parkin affects tumor cell metabolism (9, 12, 13). A proteomic study identified a number of enzymes in metabolism as candidate substrates for parkin (9). Parkin prevents the Warburg effect and promotes oxidative metabolism as a p53 target gene (13). However, the role of parkin in tumor cell growth inhibition remains obscure.

Glycolysis is the essential metabolism pathway for cell growth and survival. Compared with normal cells, tumor cells often have an increased rate of glycolysis and utilize much more glucose to keep the balance among the production of ATP, biosynthesis of building blocks, and reducing equivalents for rapid proliferation (14, 15). The key step of glycolysis is catalyzed by pyruvate kinase to convert phosphoenolpyruvate to pyruvate. Pyruvate kinase M2 (PKM2)^2^ is a less active isoform of pyruvate kinase and is important for tumor cell maintenance and growth (16–21). Its enzymatic activity is allosterically regulated; the natural ligands and allosteric regulators of PKM2 include fructose 1,6-bisphosphate (22), serine (23), and phosphoribosylaminoimidazolesuccinocarboxamide (24). It was reported that PKM2 is phosphorylated (25–27) and acetylated (28, 29), indicating that PKM2 can be regulated by post-translational modification.

In this study, we identified parkin as a regulator of PKM2 through biochemical purification of protein complex. Parkin is a specific PKM2-interacting protein and catalyzes ubiquitin conjugation to PKM2 mainly on sites Lys-186 and Lys-206. Ubiquitylation of PKM2 decreases its enzymatic activity. In contrast, PKM2 enzymatic activity is enhanced after ablation of parkin, hence increasing the steady state metabolite levels of glycolysis in cells. This is the first direct evidence to support the concept that parkin suppresses tumor growth by inhibiting glycolysis through PKM2 ubiquitylation.

## Experimental Procedures

### 

#### 

##### Plasmids, Antibodies, and Cell Culture

The HA-parkin plasmid was obtained from Addgene. The full-length parkin was amplified by PCR from HA-parkin and subcloned into pCin4-FLAG-HA, pCin4-FLAG, or pCMV-myc expression vectors. Parkin was subcloned into pTRIPZ lentiviral inducible vector. The full-length PKM2 was amplified by RT-PCR from human cells. The cDNA sequences corresponding to full-length parkin and different fragments of parkin and those corresponding to full-length PKM2 were amplified by PCR and subcloned into pGEX (GST) vector for expression in bacteria. Antibodies used in Western blotting assay were β-actin (A15), FLAG M2 from Sigma; HA (3F10) from Roche Applied Science; PKM2 (3198), Parkin (2132) and COX IV from Cell Signaling; citrate synthase form Abcam; Myc (9E10) from Santa Cruz.

H1299, 293T, U87 and IMR32 cells were cultured in DMEM medium (Cellgro), supplemented with 10% fetal bovine serum (Gibco). MCF10A cells were maintained in DMEM/F12 50/50 medium (Cellgro), supplemented with 5% horse serum, 20 ng/ml EGF, 10 μg/ml insulin, 100 ng/ml cholera toxin and 0.5 mg/ml hydrocortisone. The FLAG-HA-parkin/H1299 stable line or FLAG-HA-PKM2/H1299 stable line was established by transfecting H1299 cells with plasmid expressing pCin4-FLAG-HA-parkin or pCin4-FLAG-HA-PKM2, respectively. And cells were selected with 1 mg/ml G418 (EMD Biosciences). The inducible parkin/H1299 cell lines were established by transfecting H1299 cells with pTRIPZ lentiviral inducible parkin plasmid, and selected with puromycin.

Transfections with plasmid DNA or siRNA oligos were performed by Lipofectamine 2000 (Invitrogen) according to the manufacturer's protocol.

##### Western Blotting Analysis and Immunoprecipitation

For Western blotting analysis, cells were lysed in cold radioimmune precipitation assay buffer (20 mm Tris-HCl, pH 8.0, 150 mm NaCl, 1% Triton X-100, 1% deoxycholate, 0.1% SDS, 1 mm EDTA, 10% glycerol, and freshly supplemented proteinase inhibitor mixture).

The immunoprecipitation assay was performed with cell cytoplasmic extracts. Cytoplasmic extracts were prepared by resuspending cell pellet in hypotonic buffer (10 mm Tris-HCl, pH 7.9, 10 mm KCl, and 1.5 mm MgCl_2_ supplemented with fresh proteinase inhibitor mixture and 0.2% CHAPS), Dounce homogenizing (20 strokes with a Type A pestle), and pelleting nuclei (1000 × *g* for 10 min). The supernatant was kept as the cell cytoplasmic extracts. Cytoplasmic extracts were adjusted to a final concentration of 100 mm NaCl and 0.1% CHAPS.

Cell cytoplasmic extracts were incubated with parkin-specific antibody or PKM2-specific antibody at 4 °C overnight followed by Protein A/G beads for 4 h to analyze endogenous parkin or PKM2. After washing five times with BC100 buffer (20 mm Tris-HCl, pH 7.9, 100 mm NaCl, 10 mm KCl, 1.5 mm MgCl_2_, 20% glycerol, and 0.1% Triton X-100), the bound proteins were eluted by 1× SDS loading buffer with heat to denature proteins. Alternatively, cell cytoplasmic extracts were incubated with FLAG-agarose beads (Sigma) or HA-agarose beads (Roche Applied Science) at 4 °C overnight to analyze cells transfected with FLAG-tagged or HA-tagged plasmid. The beads were washed five times with BC100 buffer, and the bound proteins were eluted using FLAG peptide or HA peptide in BC100 buffer for 2 h at 4 °C.

##### Protein Complex Purification

Protein complex purification was performed as described previously (30, 31) with some modifications. The cytoplasmic extracts of the FLAG-HA-parkin/H1299 stable lines or FLAG-HA-PKM2/H1299stable lines were prepared as described above and subjected to a FLAG M2 and HA two-step immunoprecipitation. The tandem affinity-purified parkin or PKM2-associated proteins were analyzed by liquid chromatography (LC)-MS/MS.

##### GST Pulldown Assay

GST or GST-tagged fusion proteins were purified as described previously (30, 31). [^35^S]Methionine-labeled proteins were prepared by *in vitro* translation using the TnT Coupled Reticulocyte Lysate System (Promega). GST or GST-tagged proteins were incubated with ^35^S-labeled proteins at 4 °C overnight in BC100 buffer + 0.2% BSA and then incubated with GST resins (Novagen) for 4 h. The resins were washed five times with BC100 buffer. The bound proteins were eluted with 20 mm reduced glutathione (Sigma) in BC100 buffer for 2 h at 4 °C and resolved by SDS-PAGE. The pulled down ^35^S-labeled protein was detected by autoradiography.

##### Parkin Knockdown

Ablation of parkin was performed by transfecting cells with siRNA duplex oligonucleotides (On-Target-Plus Smart Pool: 1, catalog number J-003603-05; 2, catalog number J-3603-06; 3, catalog number J-3603-07; and 4, catalog number J-3603-08) from Thermo Sciences and control siRNA (On-Target-Plus-Si Control Nontargeting Pool, D00181010, Dharmacon). The cells were transfected three times.

Ablation of parkin in MCF10A cells were performed by infection with shRNA lentivirus. Parkin-specific shRNA plasmids and control shRNA plasmid were received from Thermo Sciences (1, catalog number V2LHS_84518; 2, catalog number V2LHS_84520; 3, catalog number V3LHS_327550; and 4, catalog number V3LHS_327554). The lentivirus was packaged in 293T cells and infected cells as described in the manufacturer's protocol. Ablation of parkin in U87 cells and FLAG-HA-parkin/U87 stable line was performed by transfecting cells once with a pool of four siRNA duplex oligonucleotides against parkin 3′-UTR region (1, CCAACTATGCGTAAATCAA; 2, CCTTCTCTTAGGACAGTAA; 3, CCTTATGTTGACATGGATT; 4, GCCCAAAGCTCACATAGAA).

##### Cell-based Ubiquitylation Assay

The ubiquitylation assay was performed as described previously (32) with some modification. 293 cells were transfected with plasmids expressing FLAG-PKM2, myc-parkin, and His-ubiquitin. After 24 h, 10% of cells were lysed with radioimmune precipitation assay buffer, and extracts were saved as input. The rest of the cells were lysed with phosphate/guanidine buffer (6 m guanidine-HCl, 0.1 m Na_2_HPO_4_, 6.8 mm Na_2_H_2_PO_4_, 10 mm Tris-HCl, pH 8.0, 0.2% Triton X-100, and freshly added 10 mm β-mercaptoethanol and 5 mm imidazole), sonicated, and subjected to Ni-NTA (Qiagen) pulldown overnight at 4 °C. The Ni-NTA resin-bound proteins were washed with wash buffer 1 (8 m urea, 0.1 m Na_2_HPO_4_, 6.8 mm Na_2_H_2_PO_4_, 10 mm Tris-HCl, pH 8.0, 0.2% Triton X-100, and freshly added 10 mm β-mercaptoethanol and 5 mm imidazole) once and further washed with wash buffer 2 (8 m urea, 18 mm Na_2_HPO_4_, 80 mm Na_2_H_2_PO_4_, 10 mm Tris-HCl, pH 6.3, 0.2% Triton X-100, and freshly added 10 mm β-mercaptoethanol and 5 mm imidazole) three times. The bound proteins were eluted with elution buffer (0.5 m imidazole and 0.125 m DTT) and resolved by SDS-PAGE.

To purify ubiquitylated PKM2, first all His-ubiquitin-conjugated proteins including PKM2 were purified with Ni-NTA resin as described above and eluted with elution buffer (0.5 m imidazole in BC100 buffer). The eluants were dialyzed with BC100 buffer for 16 h at 4 °C, exchanging the buffer for fresh buffer five times during that period. Then the eluants were incubated with the FLAG M2-agarose beads (Sigma) at 4 °C overnight. After washing three times with BC500 buffer (20 mm Tris-HCl, pH 7.9, 500 mm NaCl, 10 mm KCl, 1.5 mm MgCl_2_, 20% glycerol, and 0.5% Triton X-100) and two times with BC100 buffer, the bound proteins were eluted with FLAG peptide (Sigma) in BC100 buffer for 2 h at 4 °C. The ubiquitylated PKM2 proteins were dialyzed with BC100 buffer for 16 h at 4 °C and used for pyruvate kinase activity and Western blotting assays.

Another cell-based ubiquitylation assay was performed by FLAG M2 and HA tandem immunoprecipitation. 293 cells were transfected with FLAG-PKM2, myc-parkin, and HA-ubiquitin. After 24 h, the cells were lysed with BC500 buffer supplemented with proteinase inhibitor mixture, sonicated to shear chromatin, and subjected to FLAG M2 IP overnight at 4 °C. The FLAG M2 beads were washed three times with BC500 buffer and eluted with FLAG peptide in BC500 buffer. The eluants were incubated with HA-agarose beads overnight at 4 °C. After washing HA beads three times with BC500 buffer and once with BC100 buffer, the ubiquitylated PKM2 proteins were eluted with HA peptide in BC100 buffer. The proteins were subjected to Western blotting analysis with antibodies against FLAG and HA.

##### Pyruvate Kinase Activity Assay

The pyruvate kinase activity assay was performed using a pyruvate kinase activity assay kit (BioVision, catalog number 709-100) according to the manufacturer's protocol.

Cell extracts were prepared by lysing cells with 4 volumes of pyruvate assay buffer and spinning at 15,000 rpm for 15 min at 4 °C to remove insoluble material. Cell extracts or purified proteins (PKM2 or ubiquitylated PKM2) were added into a 96-well flat bottom plate. The volume was adjusted to 50 μl/well with pyruvate assay buffer. Then 50 μl of reaction mixture (46 μl of pyruvate assay buffer, 2 μl of pyruvate probe, and 2 μl of enzyme mixture) per well were added and mixed well. The absorbance *A*_570 nm_ was scanned once per minute for 40 min at room temperature. In the same time, a standard curve of nmol/well *versus A*_570 nm_ readings was plotted. Then the sample readings were applied to the standard curve to obtain the amount of pyruvate in the sample wells. The rate of pyruvate yield was normalized by the amount of total proteins in the lysate or the amount of pyruvate kinase.

##### Metabolite Analysis

Medium was removed from cells in 10-cm plates as completely as possible, and 80% methanol prechilled at −80 °C was added and incubated at −80 °C for 15 min. The cell lysate/methanol mixtures were transferred to tubes and centrifuged to remove the cell debris and proteins. The extracts were lyophilized and analyzed by mass spectrometry to identify the metabolism intermediate compounds. The parental cells were lysed with SDS, and total proteins were quantitated. The metabolite levels were obtained by LC-MS/MS, and values (given in arbitrary units) reflect the integrated peak area of an MS signal. Data were normalized by total protein content in the cells and are an average of three independent experiments. Error bars represent S.D. of the mean of triplicates. *p* values were determined by two-sample paired Student's *t* test.

## Results

### 

#### 

##### Identification of Parkin as a Unique Component of PKM2-associated Complexes

**FIGURE 1.**
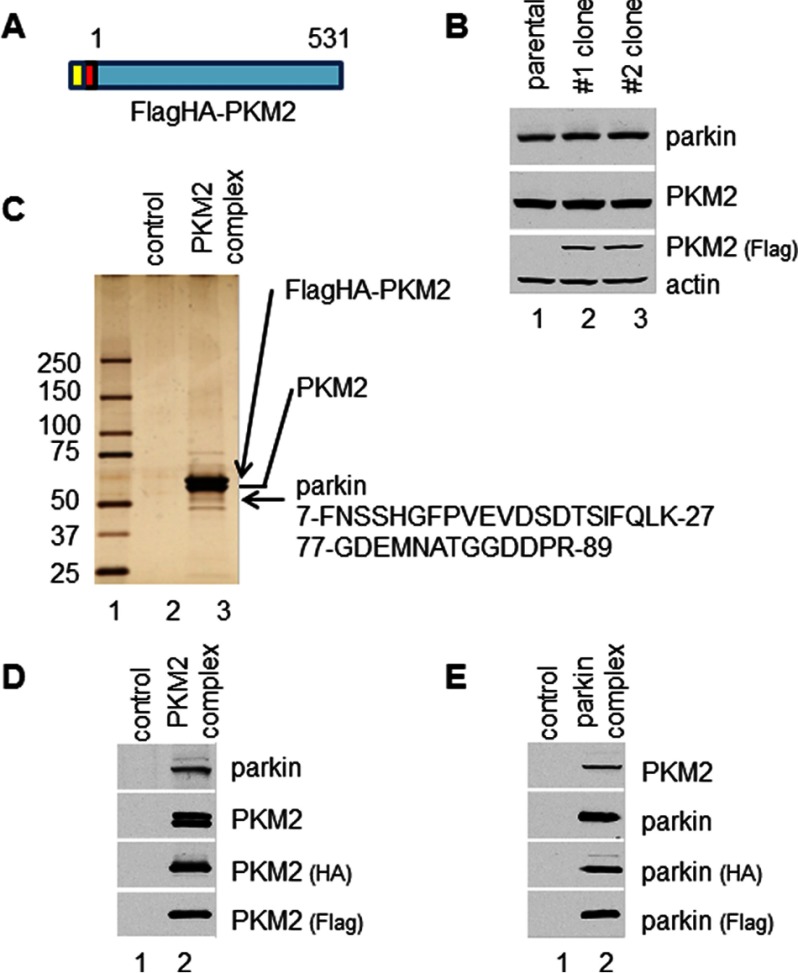
**Parkin was identified as a component of a PKM2-containing protein complex.** *A*, schematic representation of PKM2 protein used for protein complex purification. *B*, the level of FLAG-HA-PKM2 was lower than the endogenous PKM2 in the clones of the FLAG-HA-PKM2/H1299 stable cell line. The proteins were assayed with PKM2 antibody and FLAG antibody by Western blotting analysis. *C*, PKM2 protein complex was purified from the FLAG-HA-PKM2/H1299 stable cell line, resolved by SDS-PAGE, and analyzed by silver staining. The proteins in the bands of the gel were identified by LC-MS/MS. Peptide sequences identified from the LC-MS/MS analysis are presented, and bands of FLAG-HA-PKM2, PKM2, and parkin are indicated with *arrowheads. D*, Parkin was easy to detect in the PKM2 complex. PKM2 complex was assayed by Western blotting analysis with antibodies against parkin, PKM2, FLAG, and HA. *E*, PKM2 protein was in the parkin complex. The parkin protein complex was purified from FLAG-HA-parkin/H1299 stable cell line and assayed by Western blotting analysis.

To elucidate the mechanisms of PKM2-mediated cell metabolism *in vivo*, we isolated PKM2-associated protein complex from human cells. We utilize an H1299 lung carcinoma cell line that stably expresses a double-tagged human PKM2 protein with N-terminal FLAG and HA epitopes (FLAG-HA-PKM2) (Fig. 1, *A* and *B*). To isolate PKM2-containing complexes, cell cytoplasmic extracts from the stable line were subjected to two-step affinity chromatography as described previously (30, 31). The tandem affinity-purified PKM2-associated proteins were analyzed by LC-MS/MS. MS analysis revealed that two peptide sequences matched the parkin sequence in the database (Fig. 1, *C* and *D*). None of the peptide sequence of parkin was identified from the control protein complexes purified in parental H1299 cells. Parkin is likely a unique binding partner of PKM2.

To further confirm that parkin interacts with PKM2, we also established an H1299 cell line stably expressing FLAG- and HA-double tagged human parkin. We purified parkin-containing complexes from the cell cytoplasmic extracts. LC-MS/MS analysis identified two peptide sequences that matched PKM2 (Fig. 1*E*).

##### Parkin Is a Specific PKM2-interacting Protein

**FIGURE 2.**
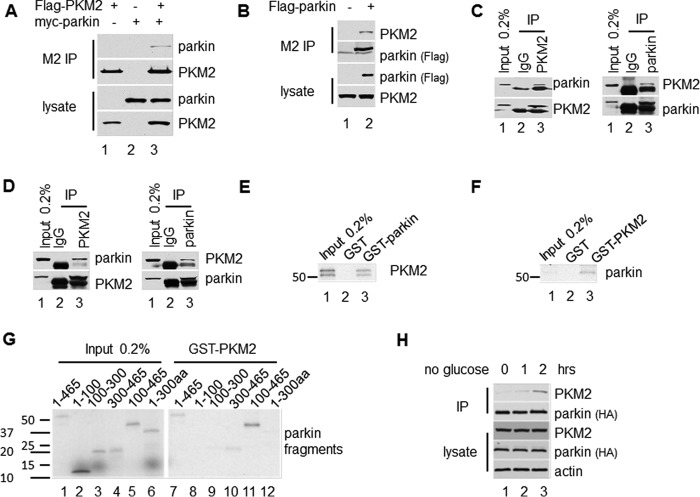
**Parkin interacts with PKM2.** *A*, H1299 cells were transfected with plasmid DNA expressing FLAG-PKM2 and/or myc-parkin. The cell extracts and the elution of IP with FLAG M2-agarose beads were assayed by Western blotting analysis. *B*, H1299 cells were transfected with plasmid DNA expressing FLAG-parkin. The cell extracts and the elution of IP with FLAG M2-agarose beads were assayed by Western blotting analysis. *C* and *D*, parkin interacts with PKM2 endogenously. Cell cytoplasmic extracts from U87 cells (*C*) or IMR32 cells (*D*) were subjected to IP with a PKM2-specific antibody or a control IgG or with a parkin-specific antibody or a control IgG. *E*, PKM2 interacts with parkin *in vitro*. The full-length GST-parkin fusion protein or GST alone was incubated with *in vitro* translated [^35^S]methionine-labeled PKM2 protein. The immobilized complex was resolved by SDS-PAGE and analyzed by autoradiography. *F* and *G*, parkin C-terminal fragment interacts with PKM2 *in vitro*. The GST-PKM2 or GST protein was incubated with *in vitro* translated [^35^S]methionine-labeled full-length parkin (*F*) or fragments of parkin (*G*). *H*, more PKM2 interacts with parkin under glucose starvation. The FLAG-HA-parkin/H1299 stable cell lines were treated without glucose for 1 and 2 h. The cell cytoplasmic extracts and the elution of IP with FLAG M2-agarose beads from cell cytoplasmic extracts were assayed by Western blotting analysis. *aa*, amino acids.

To investigate the relationship of parkin and PKM2 *in vivo*, we further examined the interaction between these two proteins. We first transiently transfected 293 cells with expression plasmids for FLAG-tagged PKM2 and myc-tagged parkin. Cell cytoplasmic extracts were subjected to immunoprecipitation with FLAG M2-agarose beads. Western blotting analysis showed that parkin is clearly detected in PKM2-associated immunoprecipitates (Fig. 2*A*). Then we transiently transfected H1299 cells with expression plasmid for FLAG-tagged parkin. Western blotting analysis revealed that endogenous PKM2 is easily detected in parkin-containing immunoprecipitates (Fig. 2*B*).

To investigate the interaction between endogenous parkin and PKM2 proteins, cytoplasmic extracts from U87 (Fig. 2*C*) and IMR32 (Fig. 2*D*) cells were immunoprecipitated with PKM2-specific antibody and control IgG or parkin-specific antibody and control IgG. As expected, parkin is easily detected in the immunoprecipitates obtained with the PKM2 antibody but not the control IgG (Fig. 2, *C* and *D*). Vice versa, PKM2 is readily detected in the parkin antibody immunoprecipitates but not the control IgG (Fig. 2, *C* and *D*). These results confirmed that endogenous parkin and PKM2 interact in cells.

To investigate the direct interaction between parkin and PKM2, we performed an *in vitro* GST pulldown assay. Purified recombinant GST-tagged parkin protein was incubated with *in vitro* translated [^35^S]methionine-labeled PKM2. Following immobilization with GST resins and recovery of captured complexes using reduced glutathione, the eluted complexes were resolved by SDS-PAGE and analyzed by autoradiography. ^35^S-Labeled PKM2 bound immobilized GST-tagged full-length parkin but not the control GST (Fig. 2*E*). Parkin was divided into different fragments according to its four domains: 1–100 fragment containing the ubiquitin-like domain, 100–300 fragment containing RING 1 domain, and 300–465 fragment containing the IBR and RING 2 domains. Purified full-length GST-PKM2 fusion proteins or GST alone was incubated with *in vitro* translated [^35^S]methionine-labeled full-length parkin protein (Fig. 2*F*) or different parkin fragments (Fig. 2*G*). The immobilized complexes were resolved by SDS-PAGE and analyzed by autoradiography. GST-PKM2 interacts with ^35^S-labeled full-length parkin and 100–465 fragment that contains RING 1-IBR-RING 2 domains (Fig. 2, *F* and *G*).

To investigate the dynamic process of the interaction between parkin and PKM2 under the condition of glucose starvation, we subjected the FLAG-HA-parkin/H1299 stable line to glucose starvation for 0, 1, and 2 h, respectively. The cytoplasmic extracts were immunoprecipitated with FLAG M2-agarose beads. Western blotting analysis revealed that parkin binds much more PKM2 protein under glucose starvation (Fig. 2*H*). Notably, although the level of PKM2 and parkin does not change, PKM2 binding by parkin was significantly enhanced upon glucose starvation. These data demonstrated that the interaction between parkin and PKM2 is regulated by the level of glucose.

##### Parkin Ubiquitylates PKM2

**FIGURE 3.**
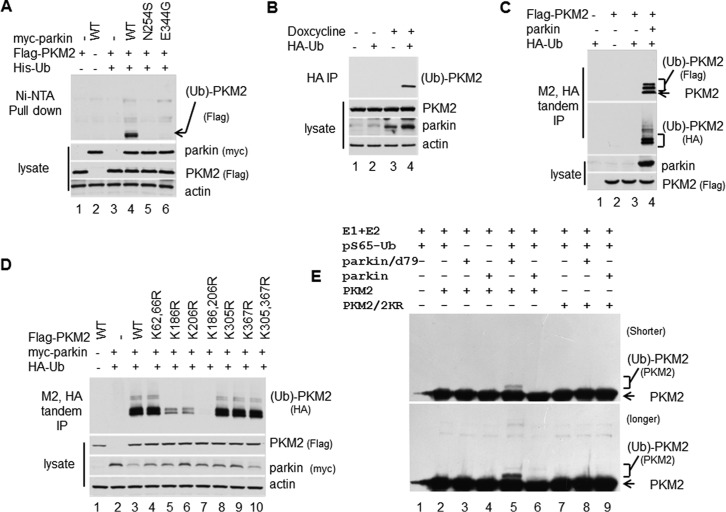
**Parkin ubiquitylates PKM2.** *A*, 293 cells were transfected with plasmid DNA expressing His-Ub, FLAG-PKM2, and/or wild type myc-parkin or mutant myc-parkin (12). The cell extracts and the Ni-NTA-agarose bead pulldown were assayed by Western blotting analysis. *B*, the inducible parkin/H1299 stable cell lines were transfected with plasmid DNA expressing HA-Ub and induced to overexpress parkin by treatment with doxycycline. The cell extracts and the elution of IP with HA-agarose beads were assayed by Western blotting analysis. *C*, 293 cells were transfected with plasmid DNA expressing FLAG-PKM2, HA-Ub, and/or parkin. The cell extracts were subjected to tandem IP with FLAG M2- and HA-agarose beads. The cell extracts and the elution of IP were assayed by Western blotting analysis. *D*, parkin ubiquitylates PKM2 mainly on Lys-186 and Lys-206. PKM2 mutants of the candidate modification sites were assayed in the same way as in *C. E*, PKM2 was ubiquitylated by the constitutively active parkin (33–35) *in vitro*. PKM2 and PKM2/K186R,K206R (labeled as *PKM2/2KR*), parkin, parkin deleted of the N-terminal 79 amino acids (labeled as *parkin/d79*), were purified with FLAG M2-agarose beads under stringent condition from the 293 cells transfected with plasmids. E1, E2, and phosphorylated Ser-65 (*pS65*) ubiquitin were purchased from Boston Biochemical.

**FIGURE 4.**
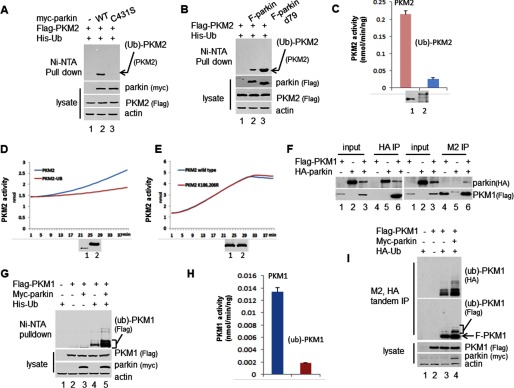
**PKM2 ubiquitylated by parkin has reduced pyruvate kinase activity.** *A*, 293 cells were transfected with plasmid DNA expressing His-Ub, FLAG-PKM2, and/or wild type myc-parkin or mutant myc-parkin/C431S (33–35). The cell extracts and the Ni-NTA-agarose bead pulldown were assayed by Western blotting analysis. *B*, 293 cells were transfected with plasmid DNA expressing His-Ub, FLAG-PKM2, and/or wild type FLAG-parkin or FLAG-parkin/d79 (33–35). The cell extracts and the Ni-NTA-agarose bead pulldown were assayed by Western blotting analysis. *C*, the activity of ubiquitylated PKM2 was reduced. The ubiquitylated PKM2 was purified by Ni-NTA-agarose bead pulldown, and then IP with FLAG M2-agarose beads under stringent condition from the 293 cells transfected with plasmids expressing FLAG-PKM2, His-Ub, and parkin was performed. The PKM2 activity was analyzed with a pyruvate kinase activity assay kit. The sample *A*_570 nm_ readings were applied to the standard curve to obtain the amount of pyruvate in the sample wells. Data represent the rate of pyruvate yield, which was normalized by the amount of the pyruvate kinase proteins. *Error bars* represent S.D. of the mean from triplicates. *D*, PKM2-Ub fusion protein to mimic monoubiquitylated PKM2 reduces PKM2 activity. PKM2 and PKM2-Ub fusion protein were purified by IP with FLAG M2-agarose beads from 293 cells transfected with FLAG-PKM2 or FLAG-PKM2-Ub plasmid. The rate of pyruvate yield in PKM2 is steeper than in PKM2-Ub; even the amount of PKM2-Ub protein is much more than the amount of PKM2, which were determined by Western blotting analysis. *E*, PKM2 mutant K186R,K206R (*K186,206R*) does not affect enzymatic activity. The rates of pyruvate yield in PKM2 wild type and mutant were the same under the same amount of proteins, which were determined by Western blotting analysis. *F*, PKM1 interacts with parkin. H1299 cells were transfected with plasmid DNA expressing FLAG-PKM1 and HA-parkin. The cell extracts and the elution of IP with FLAG M2-agarose beads or HA beads were assayed by Western blotting analysis. *G*, PKM1 was ubiquitylated by parkin. 293 cells were transfected with plasmid DNA expressing His-Ub, FLAG-PKM and myc-parkin. The cell extracts and the Ni-NTA-agarose bead pulldown were assayed by Western blotting analysis. *H*, the activity of ubiquitylated PKM1 was reduced. The ubiquitylated PKM1 was purified by Ni-NTA-agarose bead pulldown, and then IP with FLAG M2-agarose beads under stringent condition from the 293 cells transfected with plasmids expressing FLAG-PKM1, His-Ub, and parkin was performed. The PKM1 activity was analyzed with a pyruvate kinase activity assay kit. *I*, 293 cells were transfected with plasmid DNA expressing FLAG-PKM1 (*F-PKM1*), HA-Ub, and/or parkin. The cell extracts were subjected to tandem IP with FLAG M2- and HA-agarose beads. The cell extracts and the elution of IP were assayed by Western blotting analysis.

To investigate the relationship of parkin and PKM2, we examined whether parkin can ubiquitylate PKM2. 293 cells were transiently transfected with plasmids expressing His-ubiquitin (Ub), FLAG-PKM2, and parkin. Western blotting analysis showed that monoubiquitin-conjugated PKM2 bound Ni-NTA resin with wild type parkin but not with mutant parkin (12) (Fig. 3*A*). The structure of parkin was determined recently (33–35). We performed the same assay and found that an inactive mutant parkin whose active site cysteine was mutated to serine (C431S) lost its activity to PKM2 (33–35) (Fig. 4*A*), and the constitutively active parkin in which the N-terminal autoinhibitory domain was removed (parkin/d79) (33–35) had stronger activity (Fig. 4*B*). We also transfected inducible parkin H1299 stable lines with HA-Ub plasmid and induced the stable lines to express a high level of parkin by treatment with doxycycline. Western blotting analysis revealed that monoubiquitin-conjugated PKM2 was easily detected in HA immunoprecipitates of cell extracts from the cells induced to express parkin (Fig. 3*B*).

To confirm that parkin ubiquitylates PKM2, we performed a FLAG M2 and HA double immunoprecipitation assay. 293 cells were transiently transfected with plasmids expressing FLAG-PKM2, HA-Ub, and parkin. Cell extracts were subjected to a FLAG M2 and HA tandem immunoprecipitation under stringent condition. Western blotting analysis showed that monoubiquitin-conjugated PKM2 was readily detected by antibodies against FLAG and HA (Fig. 3*C*).

To identify the modification sites of PKM2, we purified ubiquitylated PKM2 proteins by FLAG M2 and HA double IP as in Fig. 3*C*. The proteins were analyzed by LC-MS/MS, and Lys-62, Lys-66, Lys-186, Lys-206, Lys-305, and Lys-367 were identified as the candidate modified sites. We further mutated PKM2 on those sites to arginine and performed a ubiquitylation assay. The results showed that the major ubiquitylation sites on PKM2 are located at Lys-186 and Lys-206 (Fig. 3*D*).

To further confirm that parkin ubiquitylates PKM2, we performed an *in vitro* ubiquitylation assay. We purified PKM2, parkin, and the constitutively active parkin (parkin/d79) from transfected 293 cells under stringent condition. The results showed that the constitutively active parkin can catalyze PKM2 ubiquitylation with phosphorylated Ser-65 ubiquitin *in vitro* (Fig. 3*E*).

##### Ubiquitylated PKM2 Attenuates PKM2 Activity

To understand the role of PKM2 ubiquitylation, we examined whether ubiquitylation of PKM2 has an effect on its enzymatic activity. 293 cells were transiently transfected with plasmids expressing His-Ub, FLAG-PKM2, and parkin. All His-Ub-conjugated proteins from cell extracts were obtained by Ni-NTA pulldown, and His-Ub-conjugated PKM2 was further purified by FLAG M2 immunoprecipitation. The pyruvate kinase activity of PKM2 and Ub-conjugated PKM2 was assessed, and although the amount of proteins was almost equal, the activity of ubiquitylated PKM2 was significantly lower than that of PKM2 (Fig. 4*C*). These data demonstrated that ubiquitin-modified PKM2 has decreased activity. To confirm this result, we purified PKM2 and PKM2-Ub fusion protein, which mimics the monoubiquitylated PKM2, and assessed their pyruvate kinase activity. The pyruvate kinase activity of PKM2-Ub was significantly reduced compared with wild-type PKM2 (Fig. 4*D*), although the pyruvate kinase activity of PKM2 K186R,K206R did not change compared with the wild-type PKM2 (Fig. 4*E*).

##### Parkin Does Not Affect PKM2 Stability

**FIGURE 5.**
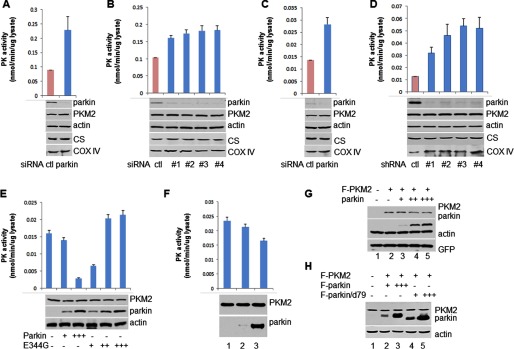
**The pyruvate kinase (*PK*) activity of the cells increases after ablation of parkin.** *A*, H460 cells were transfected with either control siRNA or a pool of four different parkin-specific siRNA oligos. *B*, H460 cells were transfected with control siRNA or four different parkin-specific siRNA oligos, respectively. *C*, MCF10A cells were transfected with either control (*ctl*) siRNA or a pool of four different parkin-specific siRNA oligos. *D*, MCF10A cells were infected with lentivirus expressing control shRNA or four different parkin-specific shRNAs, respectively. *A–D*, the cell extracts were assayed by Western blotting analysis with antibodies against parkin, PKM2, actin, citrate synthase (*CS*), and *cytochrome c oxidase* subunit IV (*COX IV*). *E* and *F*, overexpression of parkin reduces PKM2 activity. *E*, MCF10A cells were transfected with plasmid expressing wild type myc-parkin or E344G mutant myc-parkin. *F*, the inducible parkin/H1299 stable cell lines were induced to overexpress parkin. *A–F*, the pyruvate kinase activity in the cell extracts was analyzed by pyruvate kinase activity assay kit. The absorbance *A*_570 nm_ was scanned once per minute for 40 min at room temperature. Then the sample readings were applied to the standard curve to obtain the amount of pyruvate. The rate of pyruvate yield was normalized by the amount of total proteins in the lysate. Data are an average of three independent experiments. *Error bars* represent S.D. of the mean from triplicates. *G* and *H*, parkin does not degrade PKM2. *G*, H1299 cells were transfected with plasmids expressing FLAG (*F*)-PKM2 and different amounts of myc-parkin. The cell extracts were assayed by Western blotting analysis. *H*, H1299 cells were transfected with plasmids expressing FLAG (*F*)-PKM2 and different amounts of FLAG-parkin and FLAG-parkin/d79. The cell extracts were assayed by Western blotting analysis.

As shown above, parkin catalyzes ubiquitin conjugation to PKM2, so we examined whether parkin degrades PKM2 *in vivo*. Parkin was knocked down in H460 cells by transfection with the pool of four parkin-specific siRNA oligos (Fig. 5*A*) or different parkin-specific siRNA oligos (Fig. 5*B*). Although endogenous parkin was severely reduced, the level of PKM2 was unaffected. In MCF10A cells, parkin was ablated by transfection with the pool of four parkin-specific siRNA oligos (Fig. 5*C*) or by infection with different shRNA lentiviruses (Fig. 5*D*). The same as in H460 cells, the level of PKM2 remained the same. We further overexpressed parkin in cells to examine the levels of PKM2. MCF10A cells were transfected with an increasing amount of wild-type parkin or mutant parkin. Western blotting analysis showed that the level of endogenous PKM2 was stable regardless of the level of wild-type or mutant parkin overexpression (Fig. 5*E*). In an inducible parkin/H1299 cell lines, parkin was induced for overexpression, and the endogenous PKM2 remained stable (Fig. 5*F*). H1299 cells were transfected with FLAG-PKM2 and an increasing amount of myc-parkin. Western blotting analysis showed no change of the level of FLAG-PKM2 no matter how much parkin was expressed (Fig. 5*G*). H1299 cells also were transfected with FLAG-PKM2 and an increasing amount of FLAG-parkin or constitutively active FLAG-parkin (parkin/d79). The results showed that the level of FLAG-PKM2 did not change no matter how much parkin was expressed (Fig. 5*H*). These data demonstrated that parkin does not regulate PKM2 stability.

##### Parkin Interacts with PKM1 and Ubiquitylates PKM1

To investigate the relationship of parkin and PKM1, we examined the interaction between these two proteins. 293 cells were transiently transfected with expression plasmids for FLAG-tagged PKM1 and HA-tagged parkin. Cell cytoplasmic extracts were subjected to immunoprecipitation with FLAG M2-agarose beads or HA-agarose beads. Western blotting analysis showed that parkin clearly associates with PKM1 (Fig. 4*F*).

We examined whether parkin can ubiquitylate PKM1. 293 cells were transiently transfected with plasmids expressing His-Ub, FLAG-PKM1, and parkin. Western blotting analysis showed that ubiquitin-conjugated PKM1 bound Ni-NTA resin with wild-type parkin (Fig. 4*G*).

To confirm that parkin ubiquitylates PKM1, we performed a FLAG M2 and HA double immunoprecipitation assay. 293 cells were transiently transfected with plasmids expressing FLAG-PKM1, HA-Ub, and parkin. Cell extracts were subjected to a FLAG M2 and HA tandem immunoprecipitation under stringent condition. Western blotting analysis showed that ubiquitin-conjugated PKM1 was readily detected by antibodies against FLAG and HA (Fig. 4*I*).

To understand the role of PKM1 ubiquitylation, we examined whether ubiquitylation of PKM1 has an effect on its enzymatic activity. 293 cells were transiently transfected with plasmids expressing His-Ub, FLAG-PKM1, and parkin. All His-Ub-conjugated proteins from cell extracts were obtained by Ni-NTA pulldown. His-Ub-conjugated PKM1 was further purified by FLAG M2 immunoprecipitation. The pyruvate kinase activity of PKM1 and Ub-conjugated PKM1 was assessed. The activity of ubiquitylated PKM1 was significantly lower than that of PKM1 (Fig. 4*H*). These data demonstrated that ubiquitin-modified PKM1 has decreased activity.

##### Parkin Does Affect Pyruvate Kinase Activity of Cells

To further elucidate the effect of parkin on pyruvate kinase activity under physiological condition, we performed the pyruvate kinase activity assay in cells after inactivation of endogenous parkin. Parkin was knocked down in H460 cells by transfection with the pool of four parkin-specific siRNA oligos (Fig. 5*A*) or different parkin-specific siRNA oligos (Fig. 5*B*). Although endogenous parkin was severely reduced and the level of PKM2 was unaffected, the pyruvate kinase activity of cell extracts was strikingly increased (Fig. 5, *A* and *B*). In MCF10A cells, parkin was ablated by transfection with the pool of four parkin-specific siRNA oligos (Fig. 5*C*) or by infection with different shRNA lentiviruses (Fig. 5*D*). The same as in H460 cells, the level of PKM2 was unchanged, and the pyruvate kinase activity of cell extracts was strikingly increased (Fig. 5, *C* and *D*).

To further confirm the effect of parkin on pyruvate kinase activity, we performed the pyruvate kinase activity assay in parkin-overexpressing cells. MCF10A cells were transfected with increasing amounts of wild-type parkin and mutant parkin. Although Western blotting analysis showed that the level of endogenous PKM2 was unchanged, no matter how much wild-type or mutant parkin was overexpressed, the pyruvate kinase activity of cell extracts decreased with more wild-type parkin and increased with more mutant parkin (Fig. 5*E*). The inducible parkin/H1299 cells were treated with doxycycline to induce expression of a high level of parkin. The endogenous PKM2 remained stable, and the pyruvate kinase activity of cell extracts decreased with different amounts of overexpressed parkin (Fig. 5*F*). These data demonstrated that parkin regulates pyruvate kinase activity of cells.

##### Inactivation of Parkin Influences Glycolysis

**FIGURE 6.**
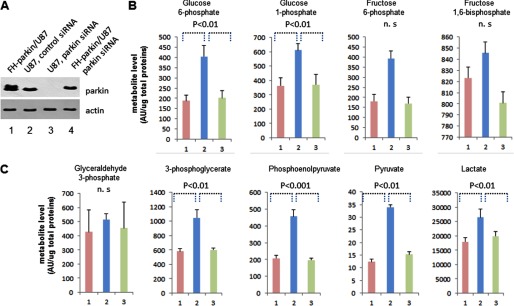
**The steady state metabolite levels of glycolysis increase after ablation of parkin.** *A*, U87 cells or FLAG-HA (*FH*)-parkin/U87 stable cell lines were transfected with either control siRNA or parkin-specific siRNA oligos. The cell extracts were assayed by Western blotting analysis. *B* and *C*, U87 cells or FLAG-HA-parkin/U87 stable cell lines were transfected with either control siRNA or parkin-specific siRNA oligos and cultured with fresh medium for 24 h. The cell metabolism intermediates were extracted with cold 80% methanol. The parental cells were lysed with SDS, and the total proteins were quantitated. *Column 1* represents U87 cells transfected with control siRNA, *column 2* represents U87 cells knocked down for endogenous parkin, and *column 3* represents FLAG-HA-parkin/U87 stable cell lines knocked down for endogenous parkin. The metabolite levels were obtained by LC-MS/MS, and values depicted in arbitrary units that reflects the integrated peak area of an MS signal. Data were normalized by total protein content in the cells and are an average of three independent experiments. *Error bar* represent S.D. of the mean from triplicates. *p* values were determined by two-sample paired Student's *t* test. *n.s.*, not significant.

To investigate the physiological function of regulation of PKM2 by parkin, we examined whether inactivation of endogenous parkin has an effect on the cell metabolic pathway. U87 cells or FLAG-HA-parkin/U87 stable cell lines were transfected once with the pool of four siRNA oligos specific for parkin 3′-UTR region or control siRNA oligos. The cells were cultured in DMEM for 24 h. The metabolism intermediate products were extracted with 80% cold methanol and analyzed by mass spectrometry. The mass spectrometric peak strength of each compound was normalized to the amount of total proteins. As shown in Fig. 6, the steady state metabolite levels of glycolysis increase after ablation of parkin and are rescued by additional FLAG-HA-parkin. Cells in the absence of endogenous parkin have higher levels of glucose 6-phosphate, fructose 6-phosphate, 3-phosphoglycerate, phosphoenolpyruvate, and pyruvate than the parental cells. However, the levels of fructose 1,6-bisphosphate, glyceraldehyde 3-phosphate, and 1,3-bisphosphoglycerate were not changed (Fig. 6). These metabolite analysis results revealed that the cells metabolic steady state levels of glycolysis are higher in the absence of endogenous parkin than in the parental cells and demonstrated that parkin plays an important role in cellular metabolism through regulating pyruvate kinase activity.

## Discussion

Parkin plays a central role in mitochondrial homeostasis and mitophagy (4). Several studies suggest that mitophagy is tumor-promoting and is required to maintain a healthy pool of mitochondria upon which tumor cells depend for growth (36, 37). However, these studies inhibited autophagy generically not just mitophagy and did not examine other aspects such as defects in turnover of endoplasmic reticulum, peroxisomes, or protein aggregates. Therefore, it is not likely that parkin functions in mitophagy to suppress tumors. Evidence has shown that defective metabolism in mitochondria instead of dysfunctional mitochondria contributes to tumor formation (38). It has been identified in several human cancers that key Krebs cycle enzymes are mutated and that metabolism in mitochondria is inherently defective (39). Studies have shown that altered expression of phosphoglycerate dehydrogenase, phosphoglycerate mutase 1, and pyruvate kinase M2 reduces the rate of glycolysis and increases biosynthetic pathways (40–44). Our results showing that parkin regulates cell metabolism through ubiquitylating PKM2 and reduces its enzymatic activity provide new evidence demonstrating the tumor suppression mechanism of parkin.

It has been shown that PKM2 is important for tumor cell survival and growth (19–24). PKM2 is necessary for aerobic glycolysis, which provides a selective growth advantage for tumor cells *in vivo* (19). PKM2 is also important for tumor cells to withstand oxidative stress and to control intracellular reactive oxygen species concentration, which are critical for tumor cell survival (23). PKM2 is functionally regulated by various post-translational modifications. Anaplastic lymphoma kinase phosphorylates PKM2 at Tyr-105, decreases PKM2 enzymatic activity, and induces cells to shift to aerobic glycolysis (25). PKM2 is also the substrate of protein-tyrosine phosphatase 1B: inhibition of PTP1B increased PKM2 Tyr-105 phosphorylation and decreased PKM2 activity. Importantly, decreased PKM2 Tyr-105 phosphorylation correlates with the development of glucose intolerance and insulin resistance in rodents, non-human primates, and humans (26). ERK1/2 also regulates PKM2 by phosphorylation at Ser-37 and converts PKM2 from a tetramer to a monomer to translocate into the nucleus. Nuclear PKM2 acts as a coactivator of β-catenin to induce c-Myc expression, resulting in the up-regulation of GLUT1, lactate dehydrogenase A, and in a positive feedback loop polypyrimidine tract-binding protein-dependent PKM2 expression and promoting the Warburg effect (27). PKM2 is also regulated by acetylation. Lys-305 acetylation under stimulation of high glucose concentration targets PKM2 for degradation through chaperone-mediated autophagy and promotes tumor growth (28). Mitogenic and oncogenic stimulation of Lys-433 acetylation, which interferes with fructose 1,6-bisphosphate binding to prevent allosteric activation, promotes PKM2 protein kinase activity and nuclear localization (29). Our findings on parkin ubiquitylating PKM2 at Lys-186 and Lys-206 and inhibiting PKM2 enzymatic activity revealed another post-translational modification event to regulate PKM2 functions and provided a new way to modulate PKM2 activity for certain cancer and Parkinson disease therapy.

Many proteins can be regulated by monoubiquitylation including histones (45), DNA replication and repair proteins (46, 47), endocytosed receptors and their regulators (48, 49), and p53 (50, 51). It has been demonstrated that monoubiquitylation works as a signal, which is recognized by ubiquitin binding domains of other proteins. Monoubiquitylation plays important roles in many biological processes such as the regulation of gene transcription, protein trafficking, and DNA repair. Many proteins can be regulated by both monoubiquitylation and polyubiquitylation with different functions. In the case of p53, low levels of Mdm2 induce p53 monoubiquitylation for p53 export from the nucleus to the cytoplasm, whereas high levels of Mdm2 polyubiquitylate p53 for proteasome degradation (50, 51). Parkin, by contrast, only induces PKM2 monoubiquitylation and decreases PKM2 activity. Inactivation of parkin increases pyruvate kinase activity followed by an increase of the steady state metabolite levels of glycolysis (Fig. 6). These results demonstrated that parkin plays important roles in cellular metabolism, and knockdown of endogenous parkin will induce in cells a metabolic shift toward aerobic glycolysis.

Pyruvate kinase M gene encodes two isoenzymes through mRNA differential splicing (19, 20). Pyruvate kinase M1 is very similar to PKM2 (only one exon, a fragment of about 20 amino acids, is different), but PKM1 is expressed in adult tissues, and PKM2 is expressed exclusively in tumors and embryos. Our results reveal that parkin regulates cell metabolism through ubiquitylating PKM2 and PKM1 and reducing their enzymatic activity. We can expect that parkin ubiquitylates PKM1 in brain, affects neuron cell metabolism, and may be the mechanism, at least in part, by which mutation of parkin causes Parkinson disease. Further work will pave a new way for therapy of Parkinson disease in the future.

## Author Contributions

K. L., F. L., and H. H. executed the experiments. Y. C. and Y. Z. executed the LC-MS/MS analysis. B. Z. executed cell metabolites analysis. W. G. and W. Z. designed, executed, analyzed, and supervised the experiments. Z. M. and J. L. analyzed the experiments. W. Z. wrote the manuscript, and J. L. helped to edit the manuscript. All authors reviewed the results and approved the final version of the manuscript.

## Footnotes

2 The abbreviations used are:

PKM: pyruvate kinase M
Ni-NTA: nickel-nitrilotriacetic acid
IP: immunoprecipitation
IBR: in between ring *fingers*
*Ub*: *ubiquitin*.
